# Hybrid oncocytic tumors (HOT) in Birt-Hogg-Dubé syndrome patients: A tale of two cities: Sequencing analysis reveals dual lineage markers capturing the two cellular populations of HOT

**DOI:** 10.1097/PAS.0000000000002152

**Published:** 2023-11-24

**Authors:** Xiao-Ming Wang, Rahul Mannan, Yuping Zhang, Anya Chinnaiyan, Roshni Rangaswamy, Seema Chugh, Fengyun Su, Xuhong Cao, Rui Wang, Stephanie L Skala, Khaled S Hafez, Ulka Vaishampayan, Jesse Mckenney, Maria M. Picken, Sounak Gupta, Reza Alaghehbandan, Maria Tretiakova, Pedram Argani, Arul M. Chinnaiyan, Saravana M. Dhanasekaran, Rohit Mehra

**Affiliations:** 1 Department of Pathology, University of Michigan Medical School, Ann Arbor, MI; 2 Michigan Center for Translational Pathology, Ann Arbor, MI; 3 Department of Urology, University of Michigan Medical School, Ann Arbor, MI; 4 Rogel Cancer Center, Michigan Medicine, Ann Arbor, MI; 5 Cleveland Clinic, Cleveland, OH; 6 Loyola pathology and laboratory medicine, Maywood, IL; 7 Mayo Clinic, Rochester, MN; 8 Department of Laboratory Medicine and Pathology, University of Washington, Seattle, WA; 9 Department of Pathology, Johns Hopkins University School of Medicine, Baltimore, MD; 10 Howard Hughes Medical Institute, Ann Arbor, MI

**Keywords:** Birt-Hogg-Dubé, hybrid oncocytic tumor, L1CAM, *LINC01187*, cell of origin, immunohistochemistry, RNA in situ hybridization, intercalated cell, kidney, hereditary renal neoplasm, mutually exclusive checkered expression pattern

## Abstract

Birt-Hogg-Dubé (BHD) syndrome is associated with an increased risk of multifocal renal tumors, including hybrid oncocytic tumor (HOT) and chromophobe renal cell carcinoma (chRCC). HOT exhibits heterogenous histological features overlapping with chRCC and benign renal oncocytoma (RO), posing challenges in diagnosis of HOT and renal tumor entities resembling HOT. In this study, we performed integrative analysis of bulk and single cell RNAseq data from renal tumors and normal kidney tissues, and nominated candidate biomarkers of HOT, L1CAM and *LINC01187*, which are also lineage-specific markers labeling the principal cell (PC) and intercalated cell (IC) lineages of the distal nephron, respectively. Our findings indicate the PC lineage marker L1CAM and IC lineage marker *LINC1187* to be expressed mutually exclusively in a unique checkered pattern in BHD associated HOT tumors, and these two lineage markers collectively capture the two distinct tumor epithelial populations seen to co-exist morphologically in HOT tumors. We further confirmed that the unique checkered expression pattern of L1CAM and *LINC01187* distinguished HOT from chRCC, RO, and other major and rare RCC subtypes. We also characterized the histopathological features and immunophenotypic features of oncocytosis in the background kidney of patients with BHD, as well as the inter-tumor and intra-tumor heterogeneity seen within HOT. We suggest that L1CAM and *LINC01187* can serve as stand-alone diagnostic markers or as a panel for the diagnosis of HOT. These lineage markers will inform future studies on the evolution and interaction between the two transcriptionally distinct tumor epithelial populations in such tumors.

## Introduction

Birt-Hogg-Dubé (BHD) syndrome is an inherited genetic disorder caused by germline mutations in the tumor suppressor gene folliculin (*FLCN*) (1). BHD is associated with an increased risk and recurrence of renal tumors, including hybrid oncocytic tumor (HOT) and chromophobe renal cell carcinoma (chRCC), which are commonly bilateral and multifocal (2, 3). HOT exhibits heterogenous histopathological and immunophenotypic features that have been reported to overlap with malignant chRCC and benign renal oncocytoma (RO). The tumor comprises clusters and individual cells with clear to eosinophilic cytoplasm and exhibits a checkerboard mosaic pattern. (4, 5) Immunohistochemical (IHC) analysis of the syndromic HOT and sporadic hybrid oncocytic/chromophobe tumor (HOCT) showed relatively similar IHC profiles to RO. (6, 7) Such overlapping histopathological and immunophenotypic features pose challenges in precise diagnosis of HOT and overlapping oncocytic renal tumor subtypes.

Molecular characterization utilizing next-generation sequencing and single cell sequencing approaches carry potential to enhance our understanding of phenotypic inter- and intra- tumor heterogeneity in renal neoplasms including HOT. This in turn helps identify candidate biomarkers to assist accurate diagnosis of related oncocytic neoplasms; such technologies also impart an enhanced insight into tumorogenesis in BHD patients, enable a cell of origin determination and help nominate diagnostic and prognostic biomarkers for renal tumors. Though molecular characterization has been recently attempted in sporadic HOCT and limited cases of syndromic HOT, no candidate biomarkers were nominated or confirmed in clinical samples. (5, 8) In our recent single cell study of renal cell cancers, we performed integrative analysis of next-generation RNA sequencing and single-cell sequencing data, identified cell of origin and nominated candidate biomarkers for various renal tumor subtypes, including chRCC and HOT (9). We characterized the expression pattern of chRCC biomarkers, FOXI1, RHCG and *LINC01187*, in a large cohort of RCC samples and confirmed these genes as lineage specific for intercalated cells (IC) of distal tubules. We also reported that all evaluated HOT cases are positive for FOXI1, RHCG, and *LINC01187* expression in a checkered staining pattern, thus indicating that one cellular component/population of HOT has an intercalated cell of origin. (10) In this study, we identified a mutually exclusive expression pattern of principal cell (PC) lineage marker, L1CAM, and intercalated cell (IC) lineage markers, *LINC01187* and FOXI1, labeling the two morphologically distinct cellular populations in HOT. We further evaluated the checkered expression pattern of L1CAM by immunohistochemistry (IHC) and *LINC01187* by RNA *in situ* hybridization (RNA-ISH) as clinical diagnostic markers in whole tissue sections representing a cohort of 111 RCC cases, including 13 HOT tumors, 3 chRCC tumors, and 2 RCC unclassified tumors from 8 BHD patients.

## Materials and Methods

### RCC cohorts

This study was performed under Institutional Review Board approved protocols with waiver for informed consent. Patient samples were obtained from the University of Michigan Medical Center, Johns Hopkins Medical Institutions, University of Washington Medicine, Loyola University Medical Center and University of British Columbia Royal Columbian Hospital. The final cohort is comprised of 18 kidney tumors from 8 genetically confirmed BHD patients (2 female and 6 male, age range 42–78, median 64) including 13 HOT, 3 chRCC, and 2 unclassified oncocytic RCC. The diagnosis of BHD associated renal tumors is based on patient medical history and clinical features associated with genetic testing for confirmation. The non-BHD renal tumors include 19 classic chRCC, 6 eosinophilic pattern of chRCC, 18 renal oncocytoma (RO), 5 low-grade oncocytic tumor (LOT), 6 oncocytic unclassified RCC, 8 clear cell RCC (ccRCC), 11 papillary RCC (PRCC), 5 clear cell papillary renal cell tumor (CCPRCT), MiTF aberration associated RCC (MiTF-RCC) including 4 *TFE3*-rearranged RCC, 1 *TFEB*-rearranged RCC and 2 *TFEB* amplified RCC, 7 eosinophilic solid and cystic renal cell carcinoma (ESC-RCC), 5 acquired cystic disease-associated RCC (ACD-RCC), 1 mucinous tubular and spindle cell carcinoma (MTSCC), and 1 succinated dehydrogenase B (SHDB) deficient RCC. In accordance with the Genitourinary Pathology Society (GUPS) descriptions for renal tumors, the term “hybrid oncocytic tumor” has been used for hereditary cases as seen in Birt-Hogg-Dubé syndrome and has no relation to the otherwise described high-grade oncocytic tumors in literature. (11)

### Biomarker nomination and lineage prediction

In our previous study, we confirmed that IC lineage markers, FOXI1 and *LINC01187*, are expressed uniformly in chRCC and renal oncocytoma (RO); we also observed ~50% of tumor cells within HOT renal neoplasms to exhibit a checkered expression pattern for these markers. To nominate markers for the HOT tumor cells not expressing IC markers, we first compared bulk RNAseq data of one BHD-HOT with average expression profile of 90 chRCC samples (10) to identify genes with high expression level in HOT and low in chRCC. To guide the lineage-specific biomarker nomination for HOT, we examined the expression signatures of benign renal tubular epithelial cell lineages from single cell RNAseq data analysis (9). After removing the low expression genes with transcript per million (TPM) <1 in HOT and chRCC, we calculated the log fold change (logFC) of each gene’s TPM in HOT compared to chRCC, and then performed pre-ranked gene set enrichment analysis (GSEA) on logFC using gene signatures (top 20 upregulated genes) of normal kidney epithelial cell types identified from scRNAseq data. (12) Top genes in the significantly enriched cell types were considered lineage biomarker candidates.

### Immunohistochemistry (IHC)

Immunohistochemistry (IHC) was performed on whole formalin fixed, paraffin embedded (FFPE) 4 micron-thick tissue sections using anti-L1CAM mouse monoclonal antibody (1:100, Abcam, catalog no. ab20148) and anti-FOXI1 rabbit polyclonal antibody (1:100, Atlas antibodies, catalog no. HPA071469). IHC was carried out on Discovery Ultra automated slide staining system (Roche-Ventana Medical Systems) using CC1 95°C for antigen retrieval, OmniMap anti-mouse HRP, and Discovery ChromoMap DAB kits (Roche-Ventana Medical Systems, Oro Valley, AZ). Dual IHC was performed consecutively with heat denaturation prior to the second primary antibody incubation using the OmniMap anti-mouse HRP and anti-rabbit HRP kits. Signals were developed using Discovery ChromoMap DAB and Red kits (Roche-Ventana Medical Systems, Oro Valley, AZ). The staining was independently assessed by three study participants including two pathologists (XWang, RMannan, and RMehra) at 100x and 200x magnification to assess for presence and pattern of expression.

### RNA in situ hybridization (RNA-ISH)

RNA *in situ* hybridization (RNA-ISH) was performed using the RNAscope 2.5 HD Brown assay (Advanced Cell Diagnostics, Newark, CA) and target probes against *LINC01187* (catalog no. 532311) as previously described (10). Dual RNA-ISH was performed using the RNAscope 2.5 HD duplex assay and target probes against *L1CAM*, *LINC01187*, and *FOXI1* (catalog no. 567451,532311-C2, and 476359-C2). RNA quality was evaluated using positive control probe targeting human housekeeping gene *PPIB*. All evaluated cases passed RNA-ISH quality control (QC) except one ESC-RCC case. Assay background was monitored using a negative control probe targeting bacterial *DapB* gene. Stained slides were examined under 100x and 200x magnification for RNA signals in tumor cells and adjacent benign kidney tissues. The RNA-ISH assay stained each RNA molecule as an individual brown, punctate dot. The staining was independently assessed by three study participants including two pathologists (XWang, RMannan, and RMehra) at 100x, 200x and 400x magnification to assess for presence and patterns of expression.

### IHC and RNA-ISH staining characterization

The staining pattern was characterized based on the presence and intensity of signals. Signals presented in equal or > 90% tumor cells was defined as uniform expression; signals presented between 75–90% tumor cells was defined as diffuse expression; signals presented in < 25% tumor cells was defined as focal expression; signals presented between 25–75% was determined to be intermediate expression.

### Statistical analysis

All statistical analyses were performed using R, v4.1.1.. Gene set enrichment analysis (GSEA) was conducted with the R package fgsea. Statistical significance was defined as a P-value <0.05.

## Results

### Nomination of lineage-specific biomarkers to distinguish the two populations of HOT

**Figure 1.**
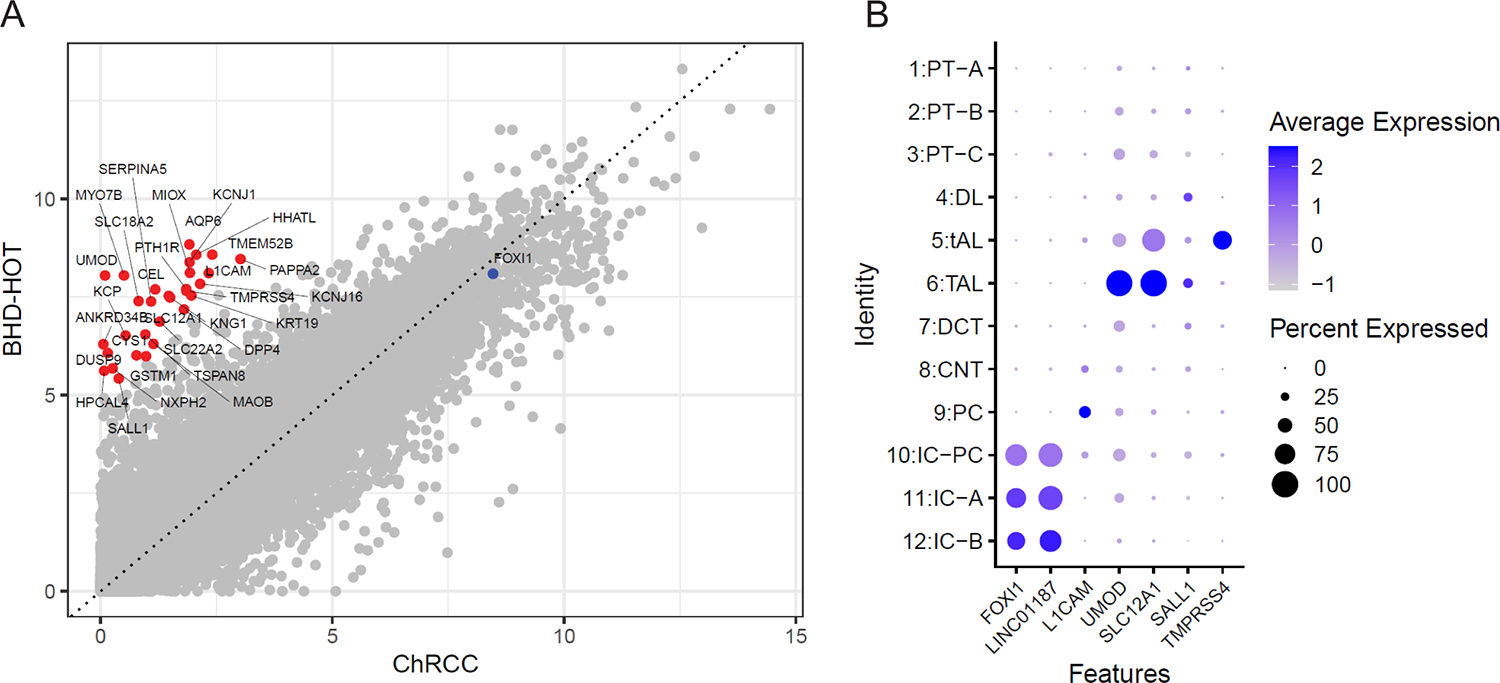
Hybrid oncocytic tumor (HOT) biomarker discovery by RNAseq and single-cell RNAseq analysis. (**A**) Correlation between HOT and chRCC bulk RNAseq data by Pearson correlation coefficient. Genes highlighted by red dots were highly expressed in HOT versus chRCC. (**B**) Dot plots showed average expression of top differentially expressed genes of HOT versus chRCC in nephron tubular epithelial cell lineages based on single-cell analysis of benign human kidney. PT-A, proximal tubule type A; PT-B, proximal tubule type B; PT-C, DL, descending limb; tAL, thin ascending limb; TAL, thick ascending limb; DCT, distal convoluted tubule; CNT, connecting duct; PC, principal cell; IC-PC, intercalated-principal cell; IC-A, intercalated cell type A; IC-B, intercalated cell type B.

In our previous study, we confirmed that intercalated cell markers, *LINC01187* and *FOXI1*, were co-expressed in one of the two cellular populations in HOT with a checkered pattern. To identify candidate markers exclusively expressed in the other HOT population, we compared the RNAseq data of 1 HOT case and 90 chRCC cases and nominated genes highly expressed in HOT, but low in chRCC, including *L1CAM*, *UMOD*, *SLC12A1*, *SALL1*, and *TMPRSS4* (Figure 1A). Based on normal human kidney scRNAseq data, *L1CAM* expression were restricted to principal cell in the distal tubules among renal cell types, *UMOD*, *SLC12A1* and *TMPRSS4* expression were enriched in thick ascending loop of Henle (TAL) (Figure 1B). GESA also confirmed significant enrichment of markers from the principal cell (PC), distal convoluted tubule cells (DCT), and thick ascending loop of Henle (TAL). (Supplementary Figure 1)

### The two cellular populations of HOT are distinct by principal cell marker L1CAM and intercalated cell marker LINC01187/FOXI1

**Figure 2.**
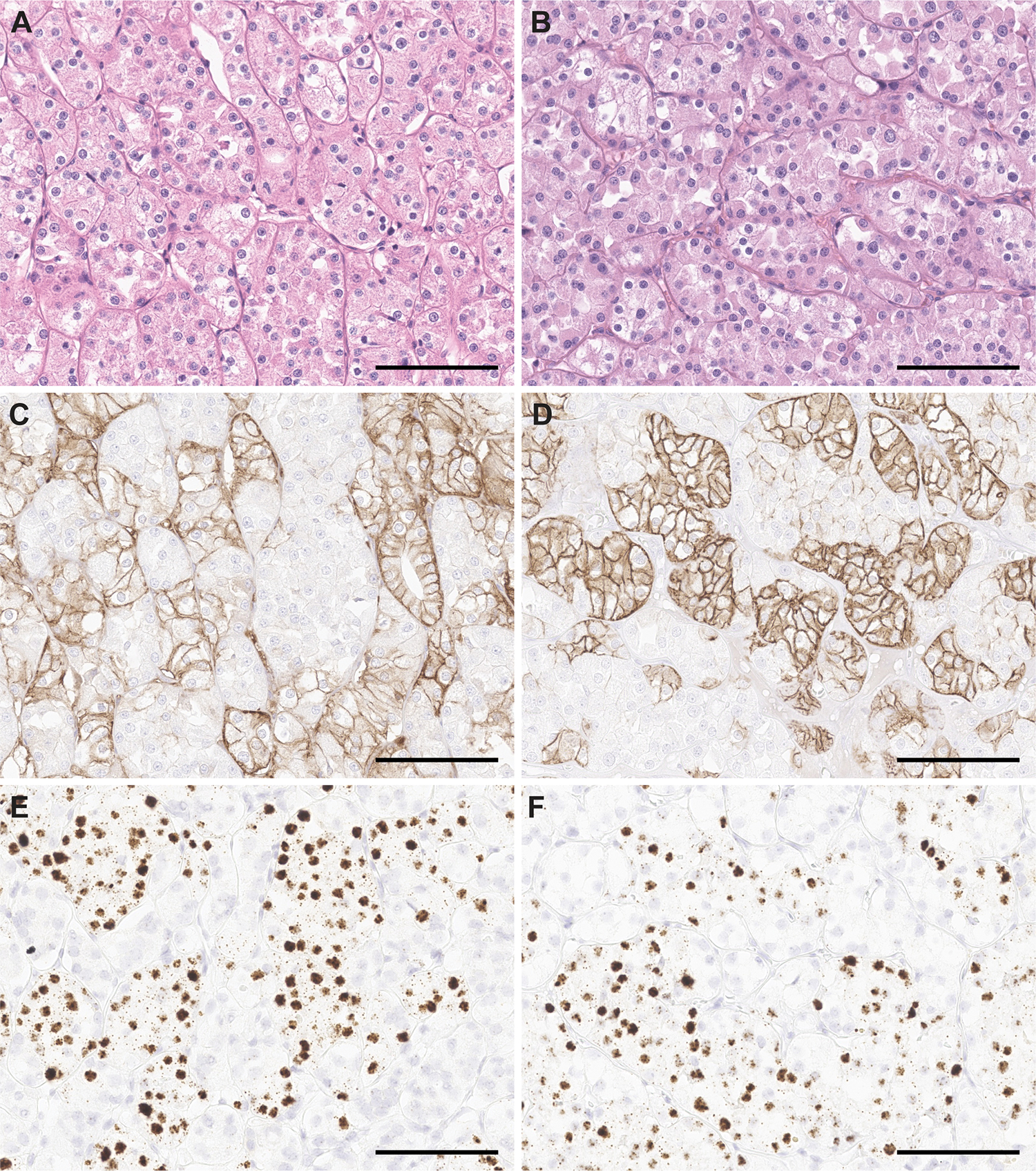
L1CAM and *LINC01187* were expressed in checkered pattern in HOT. HOT (**A, B**, H&E from two different representative cases) demonstrated strong membranous staining for L1CAM IHC (**C, D**), and nuclear staining for *LINC01187* RNA-ISH (**E, F**) in checkered pattern. Scale bars = 200μm.

**Figure 3.**
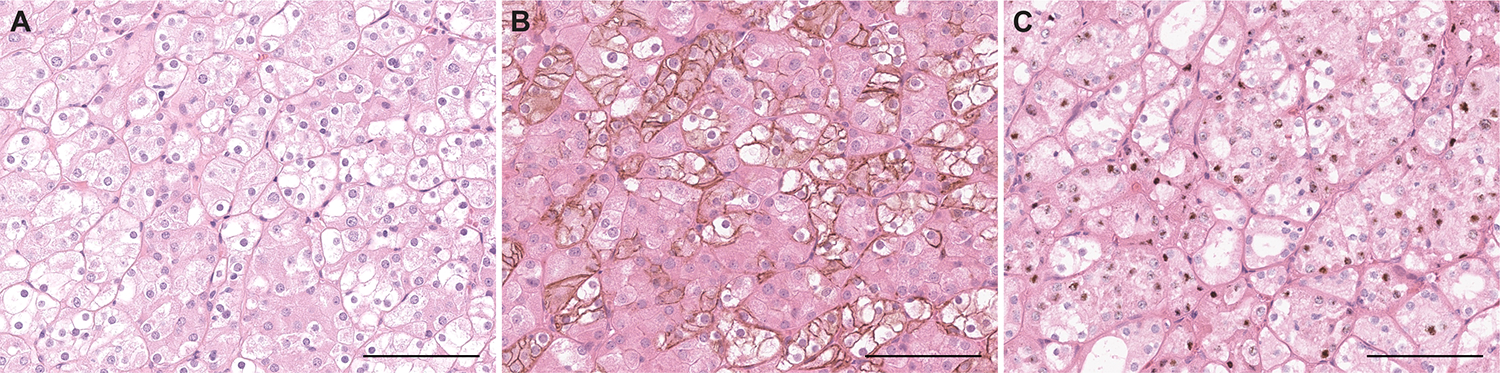
L1CAM and *LINC01187* expression were individually associated with distinct morphologies in HOT (**A**, H&E). L1CAM expression was preferentially observed in neoplastic cells with clear cytoplasm (**B**, combination of L1CAM IHC and H&E performed on the same section), and *LINC01187* expression was preferentially detected in neoplastic cells with eosinophilic cytoplasm (**C**, combination of *LINC01187* RNA-ISH and H&E performed on the same section). Scale bars = 200μm.

We performed IHC and RNA-ISH assays to confirm the expression pattern of candidate biomarkers of HOT. L1CAM IHC demonstrated strong membranous staining in one HOT population and absent to focal/low in the other HOT population, thus exhibiting a checkered pattern. Similarly, *LINC0187* RNA-ISH exhibited strong nuclear staining in checkered pattern in one HOT population and absent in the other HOT population. (Figure 2). By combining IHC/RNA-ISH staining technology with hematoxylin and eosin staining (Figure 3), we confirmed the morphologic association of the two biomarkers: the strong membranous L1CAM expression was observed in HOT tumor cells with clear cytoplasm (with a previously reported resemblance to chRCC) (Figure 3B), whereas the nuclear *LINC01187* expression was observed in HOT tumor cells with eosinophilic cytoplasm (with a previously reported resemblance to RO) (Figure 3C).

**Figure 4.**
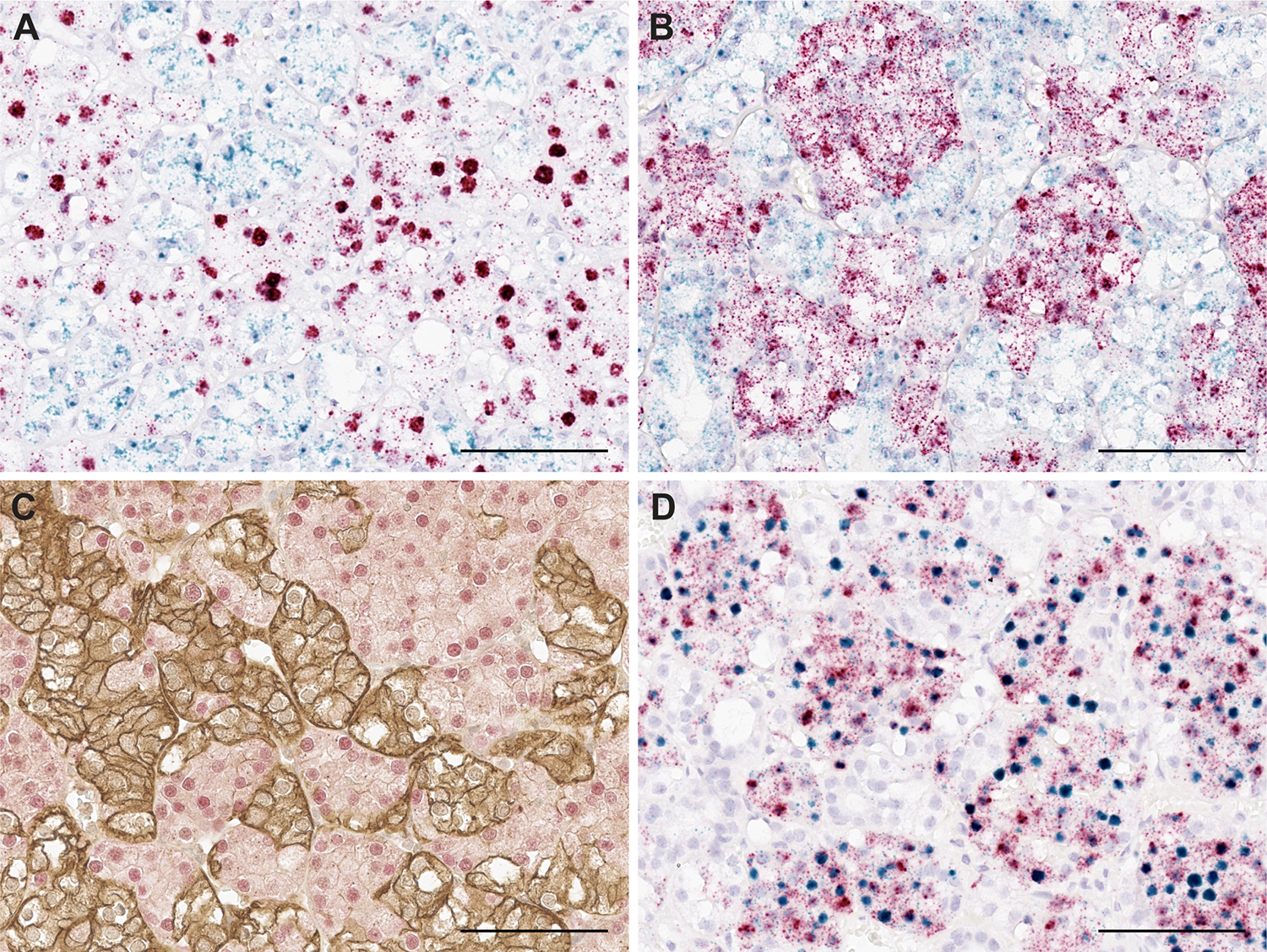
Principal cell marker L1CAM and intercalated cell marker *LINC01187*/*FOXI1* expression showed mutual exclusivity in HOT. Dual RNA-ISH for *L1CAM* (green signals) and *LINC01187* (red signals) showed mutually exclusive expression pattern in HOT (**A**). Dual RNA-ISH for *L1CAM* (green signals) and *FOXI1* (red signals) (**B**), as well as dual IHC (L1CAM-brown membranous staining and FOXI1-red signals) (**C**), also showed mutually exclusive expression pattern in HOT. Dual RNA-ISH for *LINC01187* (green signals) and *FOXI1* (red signals) showed co-expression of both genes in a cellular population of HOT emanating from the intercalated cell (**D**). Scale bars = 200μm.

Dual staining of principal cell marker L1CAM and intercalated cell markers *LINC01187*/FOXI1 confirmed that L1CAM and *LINC01187*/FOXI1 were expressed in a mutually exclusive pattern in most tumor cells in HOT. Co-detection of L1CAM and *LINC01187*/FOXI1 expression in the same cells was rare (Figure 4A–C). *FOXI1* and *LINC01187* dual RNA-ISH confirmed co-expression of both genes in one HOT population (Figure 4D).

In the adjacent benign kidney, L1CAM, FOXI1 and *LINC01187* were detected in the distal nephron segment. L1CAM was expressed in the principal cells which composed ~ 2/3 of the distal tubule cells; *LINC01187* and FOXI1 were expressed in the intercalated cells composing the remaining 1/3 of the distal tubule cells (Supplementary Figure 2).

### The unique checkered expression pattern of L1CAM and LINC01187 distinguishes HOT from chRCC and renal oncocytoma

All 13 BHD associated HOT (syndromic or BHD-HOT) and 1 of 3 BHD associated chRCC (BHD-chRCC) cases showed checkered expression of L1CAM and *LINC01187* (Table 1 & 2). Based upon the morphologic and immunohistochemical analysis performed in the current study, this BHD-chRCC case was considered to be more appropriately reclassified as a syndromic HOT. 2 BHD associated RCC unclassified cases showed diffuse expression of L1CAM and absence of *LINC01187* expression. In contrast to sporadic chRCC, which diffusely express LINC01187, 2 of 3 BHD-chRCC cases also showed diffuse expression of L1CAM and absence of *LINC01187* expression and were therefore reclassified as BHD-RCC unclassified.

18 of 19 classic chRCC cases (presenting in a sporadic fashion) had no L1CAM expression, 1 showed focal L1CAM expression, and all 19 cases exhibited uniform expression of *LINC01187* (Supplementary Figure 3A, D, G). 3 of 6 eosinophilic pattern of chRCC cases (also sporadic in nature) were positive for L1CAM expression, including 2 cases with focal expression and 1 case with single neoplastic cell positivity along with L1CAM expression in entrapped tubules. All 6 eosinophilic pattern of chRCC cases exhibited diffuse to uniform expression of *LINC01187*. (Supplementary Figure 3B, E, H) All 18 oncocytoma cases showed absence of L1CAM expression and strong uniform *LINC01187* expression. (Supplementary Figure 3C, F, I) We performed dual RNA-ISH of PC marker L1CAM and IC marker *LINC01187*/FOXI1 in classic chRCC and eosinophilic pattern of chRCC cases showing focal L1CAM expression to examine the spatial relationship of the two markers. In the examined classic chRCC cases, focal L1CAM expression were co-detected with *LINC01187* expression in the same tumor cell (Supplementary Figure 4 A, C). Distinctively, in the eosinophilic pattern of chRCC cases, focal L1CAM positive tumor cells showed no *L1NC01187* expression (mutually exclusive as in HOT). L1CAM expressing cells were quite few and with weak/focal expression in both classic chRCC and eosinophilic pattern of chRCC. (Supplementary Figure 4 B, D)

### The checkered expression pattern of L1CAM and LINC01187 distinguishes HOT from diverse oncocytic renal tumor subtypes and other RCC subtypes

We also evaluated L1CAM and *LINC01187* expression in several rare oncocytic renal tumor subtypes that could be entertained within the differential diagnosis of HOT, including LOT, oncocytic unclassified RCC, MiTF aberration associated RCC (MiTF-RCC), ESC-RCC, ACD-RCC and SDH-B deficient RCC (Table 1, Table 2, Supplementary Table 1).

All 5 LOT cases showed diffuse L1CAM expression and no *LINC01187* expression (Supplementary Figure 5). All 6 oncocytic unclassified RCC showed absence of L1CAM expression and uniform *LINC01187* expression.

We evaluated all 3 types of MiTF-RCC, including 4 *TFE3*-rearranged RCC, 1 *TFEB*-rearranged RCC, and 2 *TFEB*-amplified RCC cases. One case of each MiTF-RCC subtypes showed variable level of L1CAM expression. All MiTF-RCC cases exhibited absence of *LINC01187* expression.

In addition, all 7 evaluated ESC-RCC cases showed patchy L1CAM expression in the cystic component. 1 of 5 cases of ACD-RCC showed intermediate level of L1CAM expression with some checkered pattern observed. 1 SDHB deficient RCC case showed diffuse expression of L1CAM. Importantly, *LINC01187* expression was absent in all 6 ESC-RCC cases (1 additional ESC-RCC case failed RNA-ISH QC), 5 ACD-RCC cases, and 1 SDHB deficient RCC case evaluated. (Supplementary Table 1).

We also evaluated major RCC subtypes, ccRCC and PRCC, as well as rare RCC subtypes. L1CAM expression was absent from all ccRCC cases. 2 of 7 PRCC cases exhibited diffuse L1CAM expression, including one case of papillary renal neoplasm with reverse polarity. 1 of 4 PRCC (erstwhile called, type 2) cases and 1 MTSCC case showed focal L1CAM expression. 2 of 5 CCPRCT cases showed diffuse L1CAM expression. Importantly, *LINC01187* expression was absent from all evaluated cases of ccRCC (8 cases), PRCC (11 cases), CCPRCT (5 cases), and MTSCC (1 case) (Supplementary Table 1, Supplementary Figure 6).

### Oncocytic nodules in adjacent benign background kidney of BHD patients with HOT

**Figure 5.**
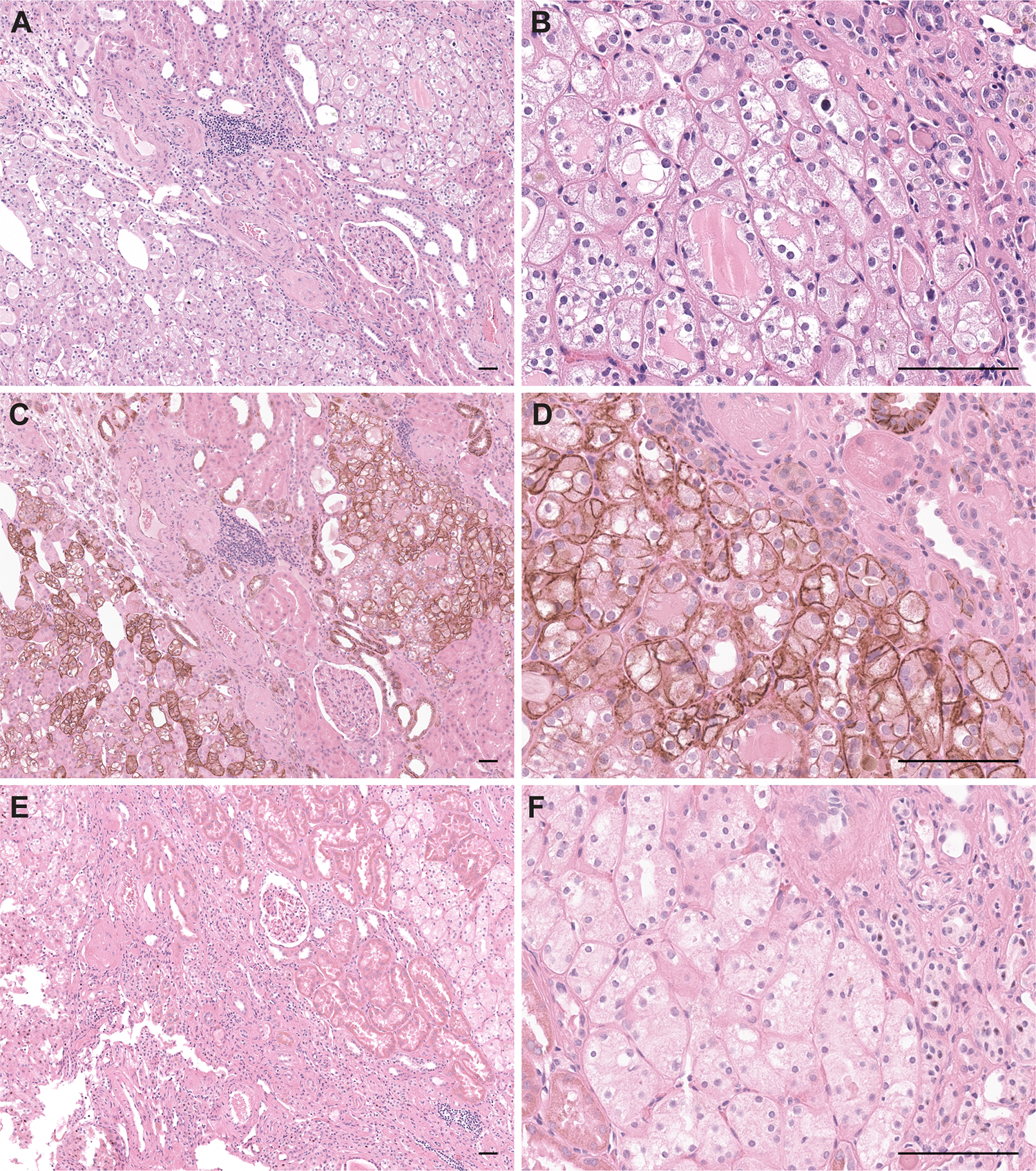
Renal oncocytosis in adjacent background renal parenchyma of patients with HOT. Benign nodules (**A, B**, H&E) in background renal parenchyma exhibited diffuse L1CAM expression (**C**, **D**, combination of L1CAM IHC and H&E performed on the same section) but no *LINC01187* expression (**E**, **F**, combination of *LINC01187* RNA-ISH and H&E performed on the same section). Scale bars = 200μm.

We evaluated for renal oncocytosis in 8 HOT cases with adjacent renal parenchyma present. Renal oncocytosis were observed in 5/8 (62.5%) HOT cases. These oncocytic nodules were composed of cells with relatively uniform cellular size, seen to be percolating in between background renal tubules. They exhibited moderate to high membranous expression of L1CAM and showed no expression of *LINC01187*; hence these immunohistochemical features were seen to be different from the immunophenotype seen within the dominant HOT mass of these patients, with a checkered expression pattern of L1CAM and *LINC01187*. (Figure 5)

### Inter-tumor and Intra-tumor heterogeneity of HOT

**Figure 6.**
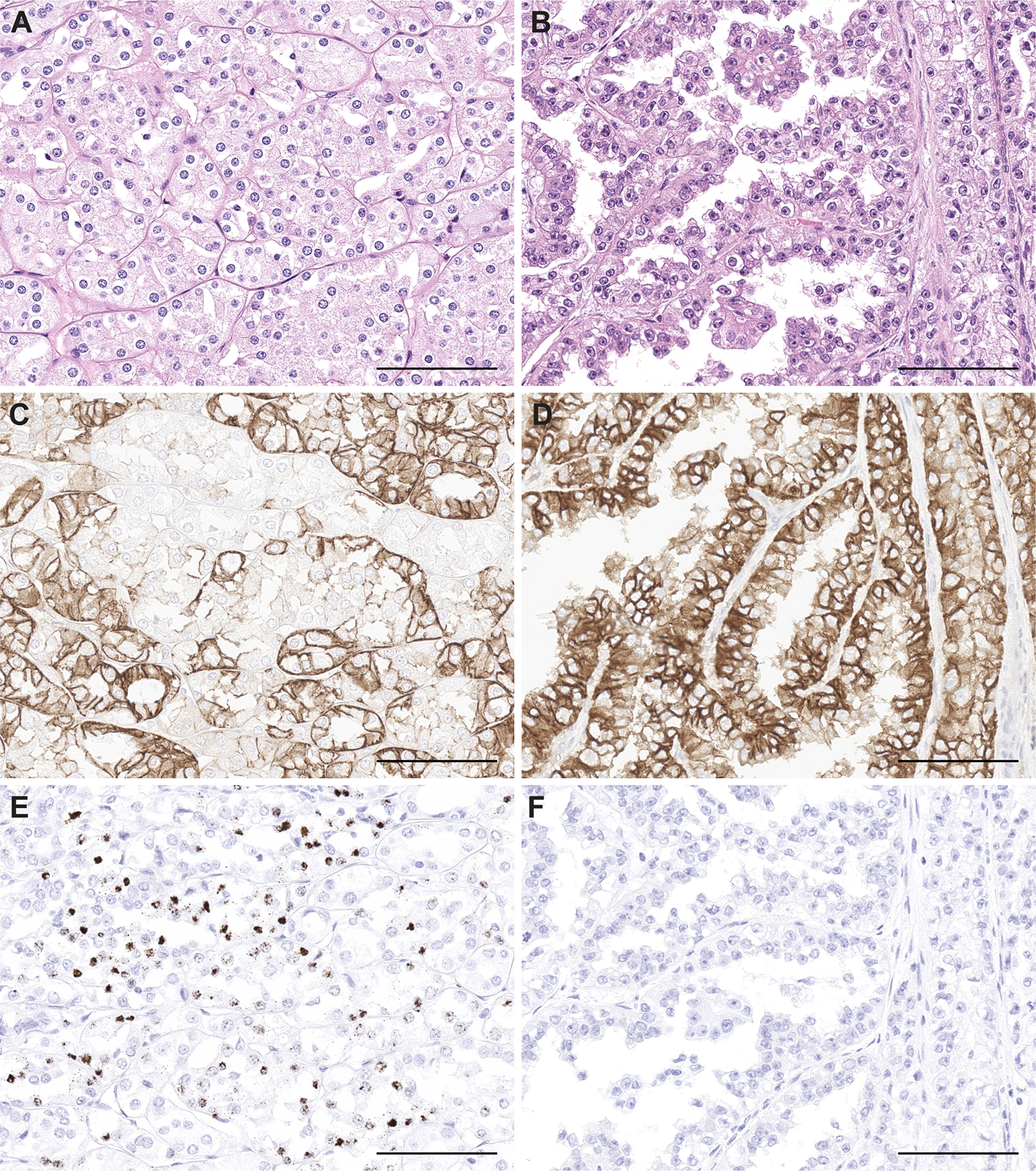
A BHD patient with multi-focal renal tumors demonstrated inter-tumor heterogeneity. In this patient with BHD, a HOT neoplasm (**A**, H&E) showed checkered expression of L1CAM (**C**) and *LINC01187* (**E**); an unclassified RCC in the same patient (**B**, H&E) showed diffuse expression of L1CAM (**D**) and no expression of *LINC01187* (**F**). Scale bars = 200μm.

BHD associated renal tumors were recurrent and multifocal. We encountered one BHD patient with 4 tumor nodules, including 2 exhibiting HOT morphology and 2 with features of unclassified RCC. The 2 HOT samples had checkered expression of both L1CAM and *LINC01187*. The 2 unclassified RCC samples had diffuse L1CAM expression and no *LINC01187* expression. This observation demonstrated the inter-tumoral heterogeneity of BHD associated renal tumors, in the axis of morphologic to molecular features. (Figure 6) We also evaluated the spatial distribution of the two HOT cellular populations within individual HOT tumor by examining the distribution of the *L1CAM* and *LINC01187* expression in a single HOT tissue section through dual RNA-ISH staining. Even though the overall ratio of *L1CAM*+ tumor and *LINC01187*+ tumor is roughly 1 to 1, we observed focal variations of *L1CAM*+ and *LINC01187*+ tumor compositions, including *L1CAM*+ dominant colonies (>75% *L1CAM*+, Supplementary Figure 7 A, B), mixed colonies (~50% *L1CAM*+ & 50% *LINC01187*+, Supplementary Figure 7 C, D), and *LINC01187*+ dominant colonies (>90% *LINC01187*+, Supplementary Figure 7 E, F) within the same tumor. This observation demonstrated the intra-tumoral heterogeneity of HOT, in terms of cellular admixture of neoplastic cells derived from intercalated cells and principal cells. Again, co-expression of *L1CAM* and *LINC01187* within the same tumor cell was a very rare event observed in HOT.

### Discussion

In this study, we established the association between phenotypic heterogeneity and molecular diversity in Birt-Hogg-Dubé syndrome associated hybrid oncocytic tumor (HOT). We performed integrative analysis of next-generation RNAseq and scRNAseq data, and nominated candidate biomarkers for HOT, including *LINC01187*, *FOXI1*, and *L1CAM*. These genes are lineage specific biomarkers of the two main distinct distal tubule cell populations, *LINC01187* and *FOXI1* for intercalated cells (IC) (9, 10, 13) and *L1CAM* for principal cells (PC) (14). All three genes were expressed in unique checkered pattern. Importantly, we reported the mutually exclusive expression pattern of the IC and PC markers in HOT, resembling the spatial relationship of IC and PC in normal kidney distal tubules.

Our results showed that both L1CAM and *LINC01187* expression can help discern HOT from other renal cell carcinomas, especially the oncocytic renal tumor subtypes with overlapping morphologies; the unique checkered staining pattern exhibited by these markers in a mutually exclusive fashion, rather than the signal intensity, is a novel and distinct biomarker expression seen in HOT from BHD patients. L1CAM IHC signals were presented in some cases of various RCC subtypes, but the unique checkered pattern in HOT is distinct, reproducible and highly distinguishable. *LINC01187* RNA-ISH demonstrated high specificity and robust signals in oncocytic tumor subtypes, including a uniform and distinct expression pattern seen in chRCC and RO compared to a checkered pattern seen in HOT. (10, 15) We suggest that L1CAM IHC serves as the first line diagnostic marker to screen for HOT, and *LINC01187* RNA-ISH as secondary marker to rule out other differential diagnosis. A combination of both L1CAM and *LINC01187* hence may be instrumental in any RCC biomarker panel for the diagnosis of HOT, and also help discern from other major and rare RCC subtypes.

In normal kidney, L1CAM is expressed in the collecting duct and connecting tubule principal cells and involved in kidney branching morphogenesis (16). L1CAM overexpression has been reported in many human carcinomas and correlated with metastasis, but rarely detected in chRCC (2%) or oncocytomas (8%) in a previous study (14). FOXI1 and *LINC01187* expression are restricted to the collecting duct intercalated cells. (9, 10, 17) FOXI1 regulates the intercalated cell fate. Loss of Foxi1 in murine models lead to failure of proper intercalated cell differentiation and disruption of acid/base transport function. (18) The function of the long noncoding RNA *LINC01187* has not yet been characterized. *LINC01187* and FOXI1 are both located on human chromosome 5q region with ~80 kilobases apart, suggesting potential transcriptional coregulation of these two genes as other characterized lncRNA-FOX family gene pairs (19, 20). The mutually exclusive expression pattern of PC and IC markers in the two morphologically distinct HOT populations suggests that principal cell and intercalated cell are the putative cell (or cells) of origin of HOT; an interplay of the resultant neoplastic mix from these two cellular compartments (or cities) results in formation of HOT.

While traditional genitourinary pathology literature review has often considered HOT within BHD patients to be constituent of cells with overlapping or admixed (morphologic) features of chRCC and RO (and hence assigned the name ‘hybrid oncocytic tumor’), this morphologic extrapolation towards nomenclature now seems to be counter intuitive. Several studies have shown both chRCC and/or RO to be of intercalated cell origin, with diffuse expression of IC cell markers like FOXI1 and *LINC01187*. If, indeed, HOT from BHD patients was comprised of cells resembling chRCC and RO, all the constituent cells should label uniformly for IC cell markers like FOXI1 and *LINC01187*. Instead, only one constituent cell within the tumor labels for IC markers; the other cellular component labels faithfully and consistently for a PC cell marker, L1CAM. Overall, our study shows that HOT is not a hybrid tumor because of admixture of ‘chRCC and RO’ like areas; instead, HOT displays morphologic and immunophenotypic heterogeneity because of an admixture of neoplastic cells arising from two distinct cells, the IC cells and PC cells. Furthermore, these markers were established and seen in almost mutually exclusive fashion (between IC and PC cell). These features support the ‘hybrid’ nature of HOT to be derived on cellular composition (from IC and PC cells), and not on basis of morphologic similarity with chRCC or RO; this dual and consistent cellular lineage comprising the originating populations for HOT, hence, now, provides a molecular and cellular rationale for continuing to use the ‘hybrid’ terminology for these tumors. Like mentioned in the above narrative, the HOT tale revolves around two distinct and separate cities (IC cells and PC cells), however, currently it is unclear how two different cells within the nephron contribute towards a distinct and unifying tumorogenesis pathway in syndromic HOT patients with *FLCN* mutations. One could speculate that a transitory cell in the physiologic state like the IC-PC cell is indeed a precursor cell to the intricate HOT tumors within BHD patients; spatial transcriptomics can hopefully provide some answers in this context.

*FLCN* is the causative gene for BHD syndrome. Deficiency of Flcn in mouse kidney were reported to promote kidney cell proliferation (21), kidney cyst, hyperplasia, and kidney tumors (3, 21–25) through activation of mTORC and AMPK signaling pathways (26, 27). Aligning the existing literature with our histologic and molecular characterization, our study illustrates that HOT is a product of PC-IC clonal expansion due to dysregulation of distal tubule structure and function, caused by the founding mutation of *FLCN*.

The presence of renal oncocytosis has been reported and histologically characterized in the benign kidney background of hybrid oncocytic tumors (28). Here, we characterized the involvement of renal oncocytosis in BHD-HOT adjacent benign tissues with histological and immunological features. Oncocytosis was observed in >50% HOT cases with diffused L1CAM expression and absence of *LINC01187* expression, which distinguishes them from the dominant HOT mass. The fact that cellular constituents of background renal oncocytosis exhibited L1CAM expression only in a diffuse fashion (and not checkered) and with negative *LINC01187* expression indicates that renal oncocytosis either acts as an early neoplastic precursor to well-developed and clinically manifest HOT, or is an independent morphologic and clinical observation in patients with BHD. Regardless, diffuse expression of L1CAM in these background oncocytic nodules can be of a useful diagnostic adjunct to renal oncocytosis in patients with BHD.

In conclusion, the principal cell marker L1CAM and intercalated cell marker *LINC01187* are expressed mutually exclusively in a unique checkered pattern in BHD associated HOT tumors. These two lineage markers collectively capture the two distinct tumor epithelial populations that co-exist in BHD associated HOT tumors, and reliably helps distinguish HOT from chRCC and RO. These markers will inform future studies on the evolution and interaction between the two transcriptionally distinct tumor epithelial populations in HOT. It is our future goal to evaluate *LINC01187* and L1CAM expression in sporadic HOCT cases.

## Supplementary Material

Supplementary Figure 1 **Supplementary Figure 1.** Enrichment of renal epithelial cell markers in BHD related HOT compared to chRCC. Top 6 genes in the leading edge of the plot were listed. NES, normalized enrichment score; PC, principal cell; DCT, distal convoluted tubule; TAL, thick ascending loop of Henle.

Supplementary Figure 2 **Supplementary Figure 2.** L1CAM and *LINC01187*/FOXI1 expression in benign renal tubules of background kidney. L1CAM (**A**, membranous IHC based staining, labeling principal cells) and *LINC01187* (**B**, nuclear RNA-ISH based staining, labeling intercalated cells) were strongly expressed in kidney distal tubules. Dual RNA-ISH staining for *L1CAM* (green signals) and *LINC01187* (red signals) showed mutually exclusive expression in an alternating fashion along kidney distal tubules (**C**). Dual RNA-ISH for *L1CAM* (green signals, labeling principal cells) and *FOXI1* (red signals, labeling intercalated cells) (**D**), as well as dual IHC (L1CAM-brown and FOXI1-red nuclear) (**E**), also showed alternating expression pattern in distal tubules. Dual RNA-ISH for *LINC01187* (green signals) and *FOXI1* (red signals) showed co-expression of both genes in distal tubules (**F**). Scale bars = 200μm.

Supplementary Figure 3 **Supplementary Figure 3.** L1CAM and *LINC01187* expression in classic chRCC, eosinophilic pattern of chRCC, and renal oncocytoma. Classic chRCC (**A**, H&E) showed absence or focal expression of L1CAM (**D**) and uniform *LINC01187* expression (**G**); majority of eosinophilic pattern of chRCC (**B**, H&E) showed none or focal expression of L1CAM (**E**) and uniform *LINC01187* expression (**H**); renal oncocytoma (**C**, H&E) showed no expression of L1CAM (**F**) and strong uniform *L1NC01187* expression (**I**). Scale bars = 200μm.

Supplementary Figure 4 **Supplementary Figure 4.** Focal expression of L1CAM was identified to co-express with *LINC01187*/*FOXI1* in chRCC, but mutually exclusive to *LINC01187*/*FOXI1* in pattern variant of chRCC. (**A**, **B**) *L1CAM* (green signals) and *LINC01187* (red signals) dual RNA-ISH in chRCC (**A**) and eosinophilic pattern of chRCC (**B**); (**C**, **D**) *L1CAM* (green) and *FOXI1* (red) dual RNA-ISH in chRCC (**C**) and eosinophilic pattern of chRCC (**D**); Scale bars = 200μm.

Supplementary Figure 5 **Supplementary Figure 5.** L1CAM and *LINC01187* expression in a LOT case. LOT (**A**, H&E) demonstrated diffuse L1CAM expression (**B**) and absence of *LINC01187* expression (**C**). Scale bars = 200μm.

Supplementary Figure 6 **Supplementary Figure 6.** L1CAM and *LINC01187* expression in ccRCC and PRCC. ccRCC (**A**, H&E) demonstrated no expression of L1CAM (**C**) or *LINC01187* (**E**). PRCC (**B**, H&E) showed no expression of L1CAM (**D**) in majority of evaluated cases and no expression of *LINC01187* in all evaluated cases (**F**). Scale bars = 200μm.

Supplemental Data File (.doc, .tif, pdf, etc.)_1

Supplemental Data File (.doc, .tif, pdf, etc.)_2 **Supplementary Figure 7.** Dual RNA-ISH for *L1CAM* (green signals) and *LINC01187* (red signals) in a single tissue section from a HOT neoplasm demonstrated intra-tumor heterogeneity. Area 1 (**A**, **B**-zoom in), Area 2 (**C**, **D**-zoom in), and Area 3 (**E**, **F**-zoom in) showed variable proportions of neoplastic cells within HOT expressing *L1CAM* (green signals) or *LINC01187* (red signals). Scale bars = 200μm.

## Figures and Tables

**Table 1.** L1CAM expression in BHD-HOT and oncocytic renal tumor types with overlapping morphologies.

Tumor Type	L1CAM IHC	
	Positivity	Pattern^#^
BHD-HOT*	14/14 (100%)	checkered
BHD-RCC unclassified^	4/4 (100%)	diffuse
chRCC	1/19 (5%)	1 focal
eochRCC^&^	3/6 (50%)	2 focal and 1 single cell positivity with entrapped tubules
Oncocytoma	0/18 (0%)	--
LOT	5/5 (100%)	diffuse
oncocytic unclassified	0/6 (0%)	--

* One BHD-HOT case was originally diagnosed as BHD-chRCC

^ Two BHD-RCC unclassified cases was originally diagnosed as BHD-chRCC

& Eosinophilic pattern of chRCC

# Diffuse: signals presented in equal or > 75% tumor cells; focal: signals presented in < 25% tumor cells; intermediate: signals presented in 25–75% tumor cells.

**Table 2.** *LINC01187* expression in BHD-HOT and oncocytic renal tumor types with overlapping morphologies.

Tumor Type	*LINC01187* RNA-ISH	
	Positivity	Pattern^#^
BHD-HOT*	14/14 (100%)	checkered
BHD-RCC unclassified^	0/4 (0%)	--
chRCC	19/19 (100%)	uniform
eochRCC^&^	6/6 (100%)	5 uniform, and 1 diffuse
Oncocytoma	18/18 (100%)	uniform
LOT	0/5 (0%)	--
oncocytic unclassified	6/6 (100%)	uniform

* One BHD-HOT case was originally diagnosed as BHD-chRCC

^ Two BHD-RCC unclassified cases was originally diagnosed as BHD-chRCC

& Eosinophilic pattern of chRCC

# Uniform: signals presented in > 90% tumor cells

## List of abbreviations

HOT: hybrid oncocytic tumor
HOCT: hybrid oncocytic/chromophobe tumor
chRCC: chromophobe renal cell carcinoma
RO: renal oncocytoma
LOT: low-grade oncocytic tumor
ccRCC: clear cell renal cell carcinoma
PRCC: papillary renal cell carcinoma
CCPRCT: clear cell papillary cell tumor
MiTF-RCC: MiTF aberration associated renal cell carcinoma
ESC-RCC: eosinophilic solid and cystic renal cell carcinoma
ACD-RCC: acquired cystic disease-associated renal cell carcinoma
MTSCC: mucinous tubular and spindle cell carcinoma
SHDB deficient RCC: succinated dehydrogenase B deficient renal cell carcinoma
IHC: immunohistochemistry
RNA-ISH: RNA *in situ* hybridization
FOXI1: forkhead box I1
LINC01187: long intergenic non-protein coding RNA 1187
L1CAM: L1 cell adhesion molecule
