# Supplementary figures and images for: Hybrid oncocytic tumors (HOT) in Birt-Hogg-Dubé syndrome patients: A tale of two cities: Sequencing analysis reveals dual lineage markers capturing the two cellular populations of HOT
Source: Am J Surg Pathol. Author manuscript; Available in PMC 2024 Feb 16. (PMC10871670; doi:10.1097/PAS.0000000000002152)

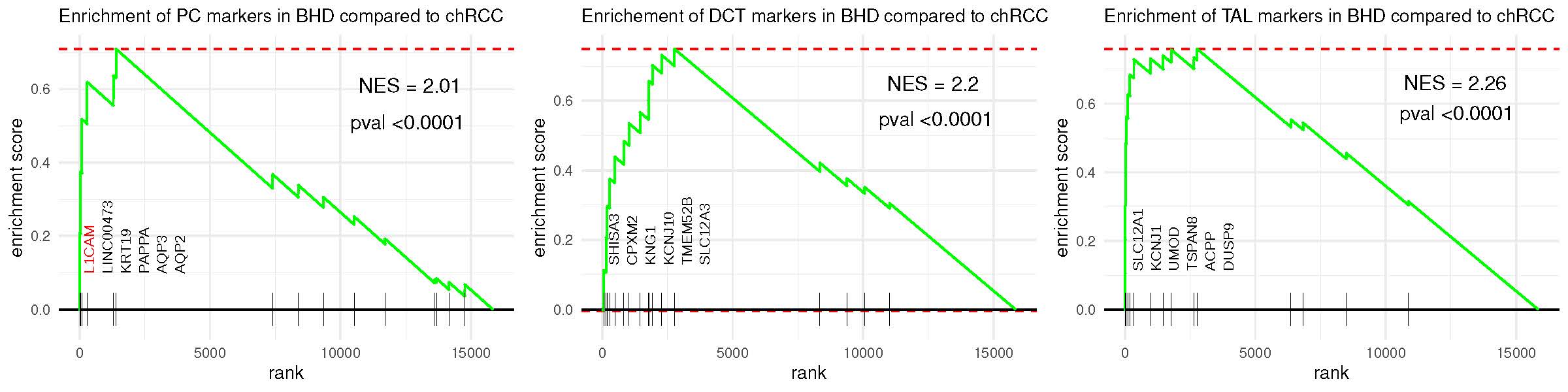

Supplement: Supplementary Figure 1 — Enrichment of renal epithelial cell markers in BHD related HOT compared to chRCC. Top 6 genes in the leading edge of the plot were listed. NES, normalized enrichment score; PC, principal cell; DCT, distal convoluted tubule; TAL, thick ascending loop of Henle. [file NIHMS1940187-supplement-Supplementary_Figure_1.jpg]

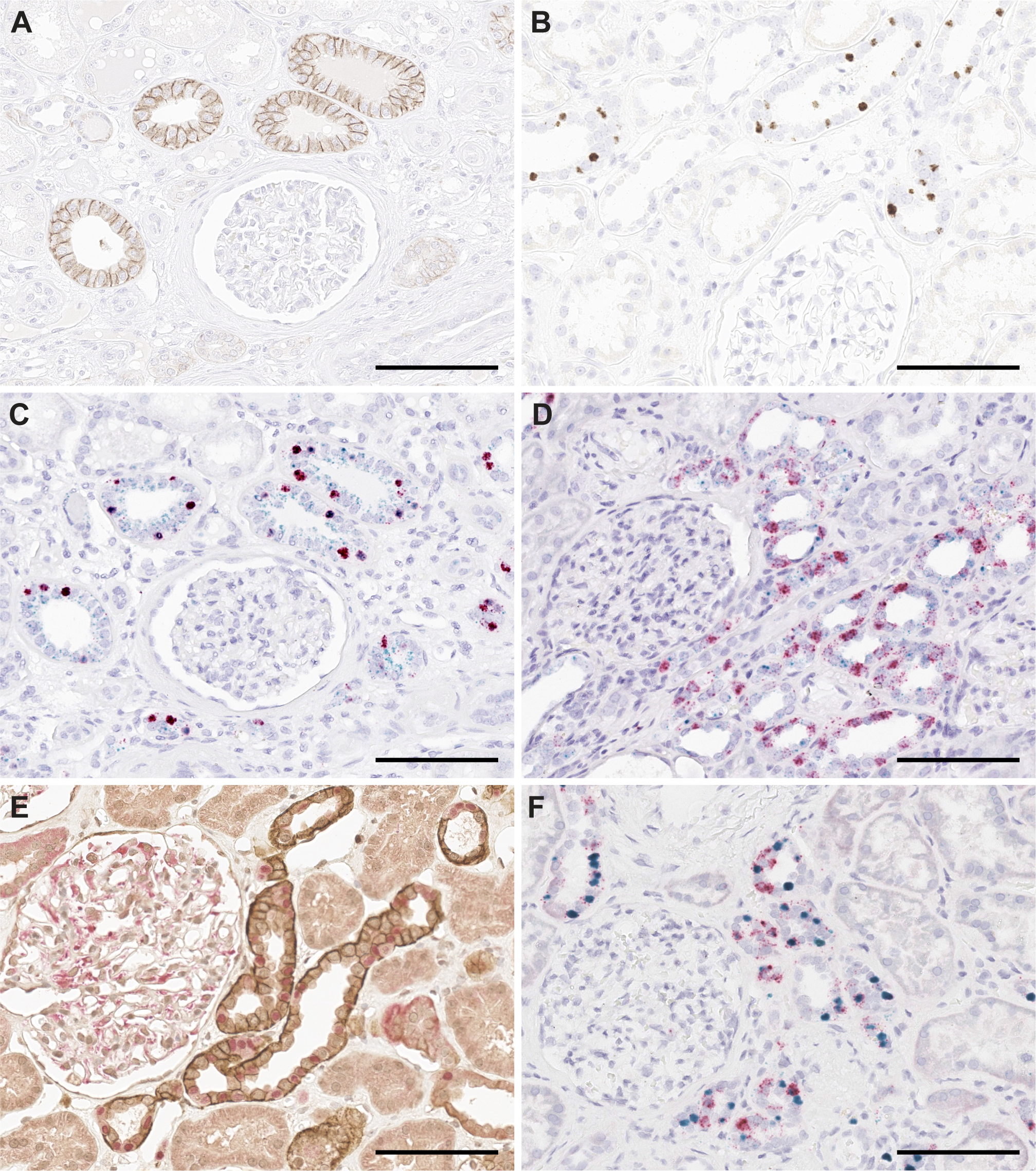

Supplement: Supplementary Figure 2 — L1CAM and LINC01187/FOXI1 expression in benign renal tubules of background kidney. L1CAM (A, membranous IHC based staining, labeling principal cells) and LINC01187 (B, nuclear RNA-ISH based staining, labeling intercalated cells) were strongly expressed in kidney distal tubules. Dual RNA-ISH staining for L1CAM (green signals) and LINC01187 (red signals) showed mutually exclusive expression in an alternating fashion along kidney distal tubules (C). Dual RNA-ISH for L1CAM (green signals, labeling principal cells) and FOXI1 (red signals, labeling intercalated cells) (D), as well as dual IHC (L1CAM-brown and FOXI1-red nuclear) (E), also showed alternating expression pattern in distal tubules. Dual RNA-ISH for LINC01187 (green signals) and FOXI1 (red signals) showed co-expression of both genes in distal tubules (F). Scale bars = 200μm. [file NIHMS1940187-supplement-Supplementary_Figure_2.tif]

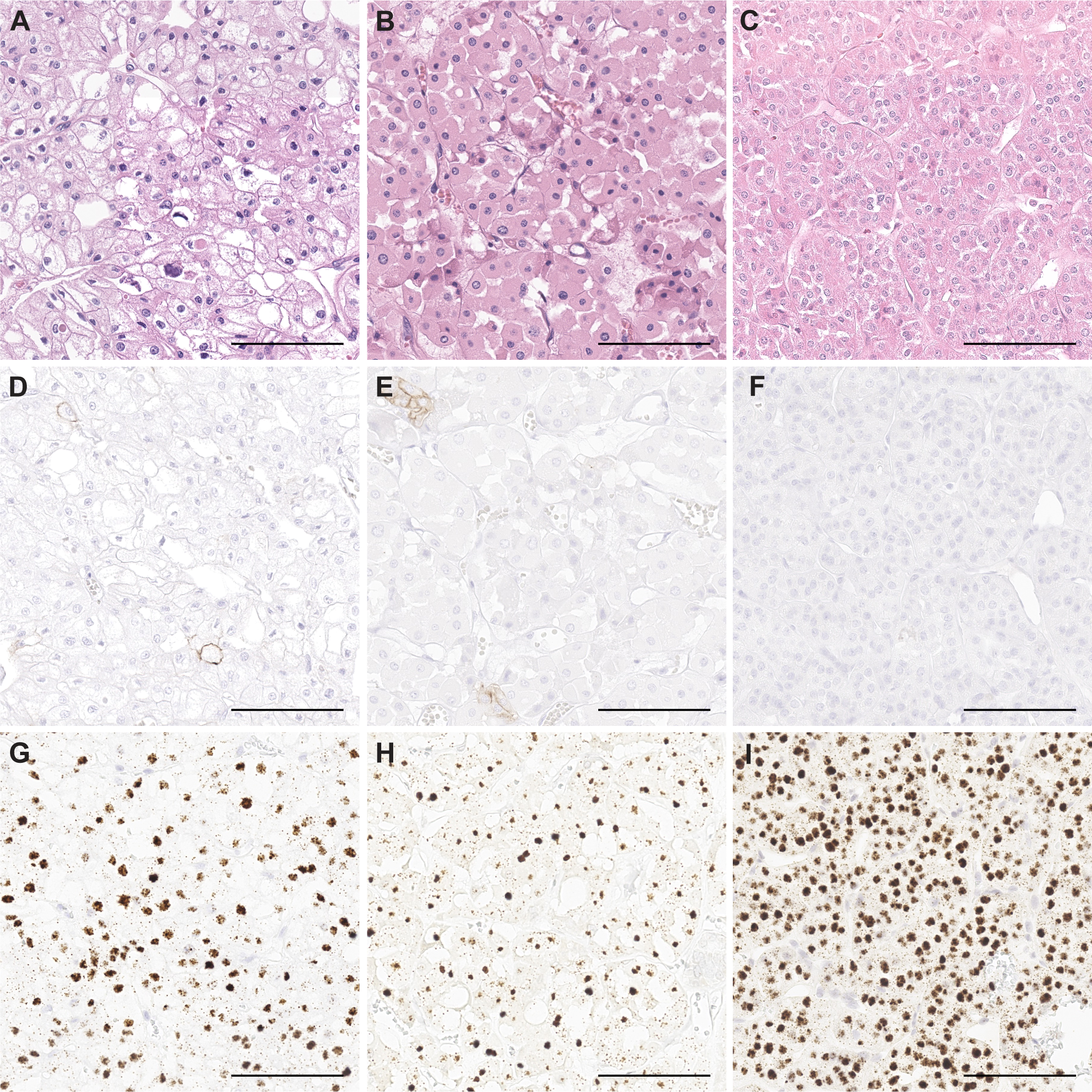

Supplement: Supplementary Figure 3 — L1CAM and LINC01187 expression in classic chRCC, eosinophilic pattern of chRCC, and renal oncocytoma. Classic chRCC (A, H&E) showed absence or focal expression of L1CAM (D) and uniform LINC01187 expression (G); majority of eosinophilic pattern of chRCC (B, H&E) showed none or focal expression of L1CAM (E) and uniform LINC01187 expression (H); renal oncocytoma (C, H&E) showed no expression of L1CAM (F) and strong uniform L1NC01187 expression (I). Scale bars = 200μm. [file NIHMS1940187-supplement-Supplementary_Figure_3.tif]

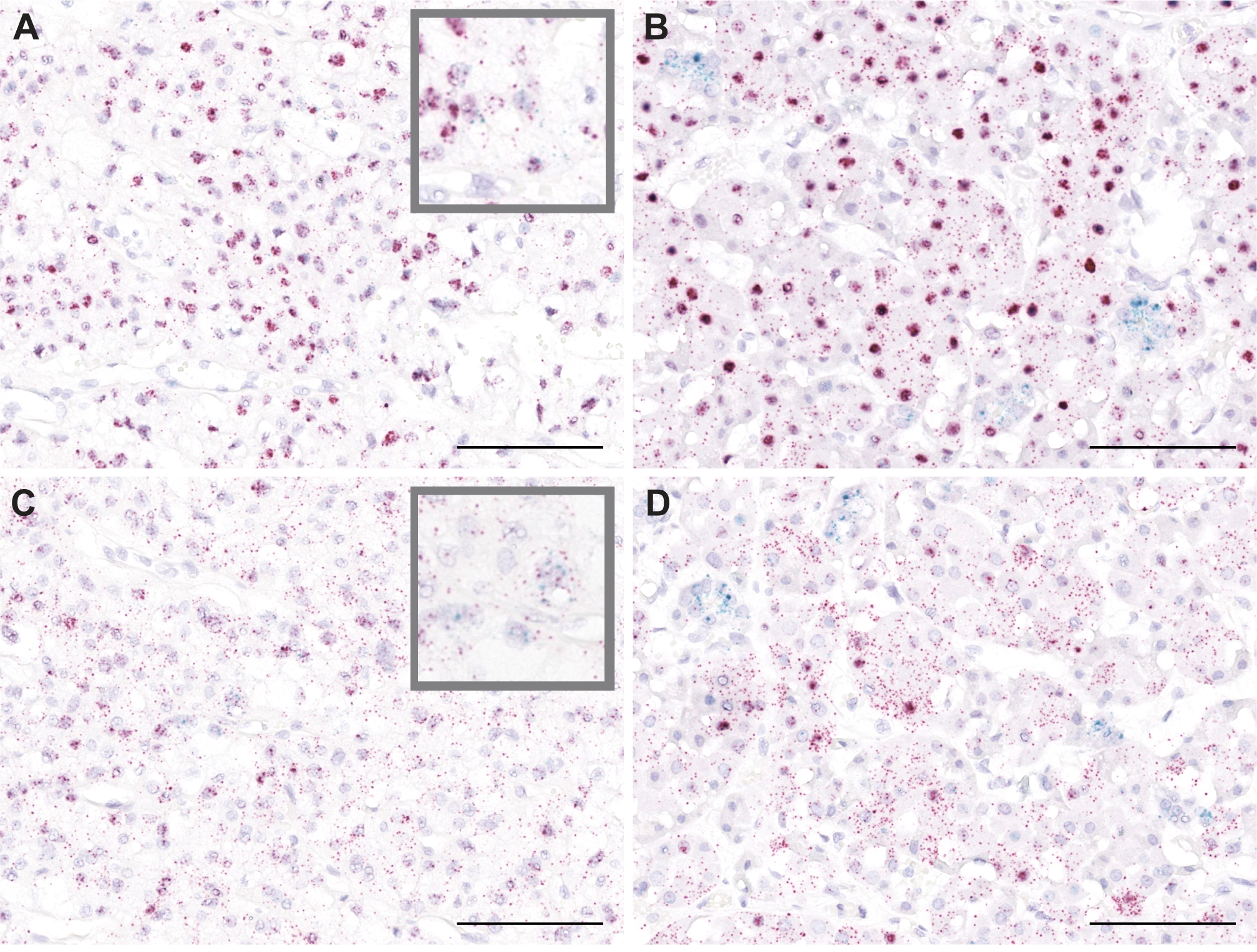

Supplement: Supplementary Figure 4 — Focal expression of L1CAM was identified to co-express with LINC01187/FOXI1 in chRCC, but mutually exclusive to LINC01187/FOXI1 in pattern variant of chRCC. (A, B) L1CAM (green signals) and LINC01187 (red signals) dual RNA-ISH in chRCC (A) and eosinophilic pattern of chRCC (B); (C, D) L1CAM (green) and FOXI1 (red) dual RNA-ISH in chRCC (C) and eosinophilic pattern of chRCC (D); Scale bars = 200μm. [file NIHMS1940187-supplement-Supplementary_Figure_4.tif]

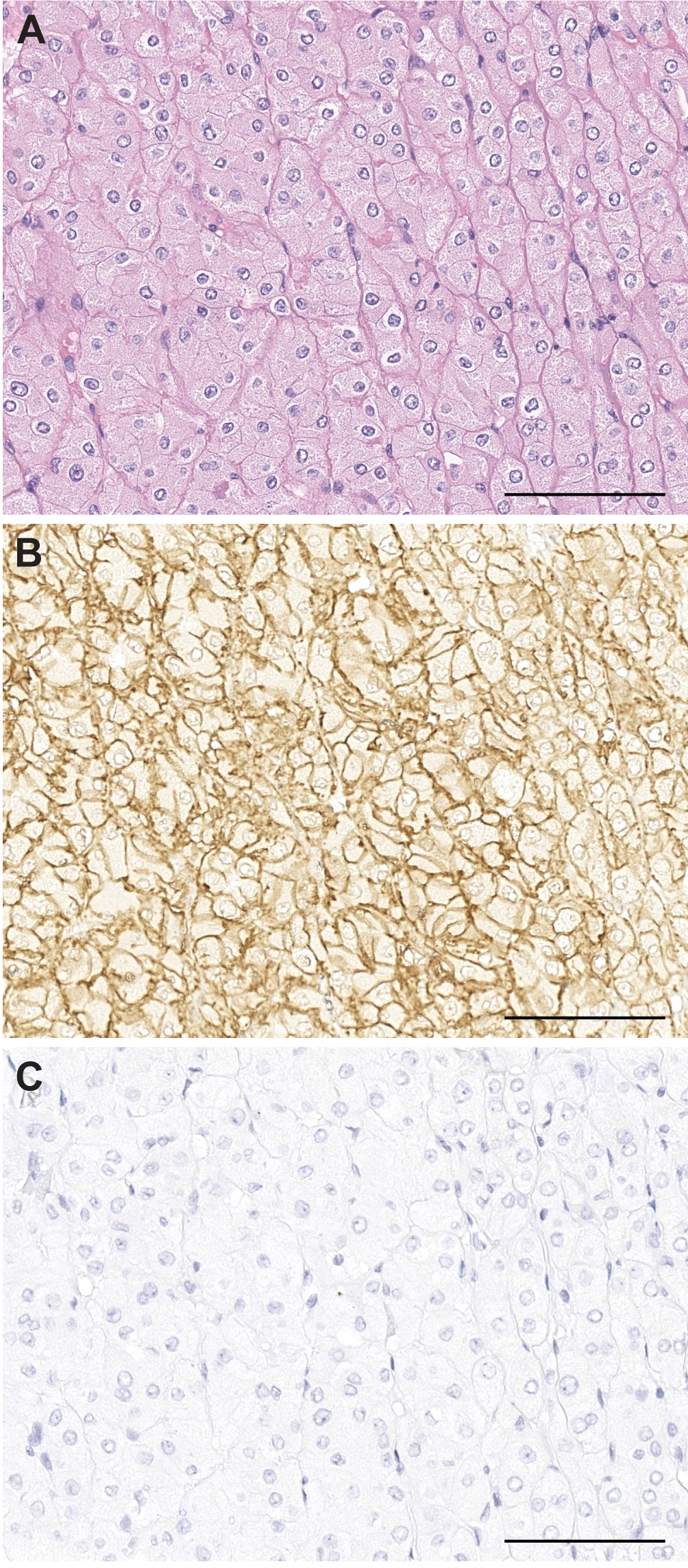

Supplement: Supplementary Figure 5 — L1CAM and LINC01187 expression in a LOT case. LOT (A, H&E) demonstrated diffuse L1CAM expression (B) and absence of LINC01187 expression (C). Scale bars = 200μm. [file NIHMS1940187-supplement-Supplementary_Figure_5.tif]

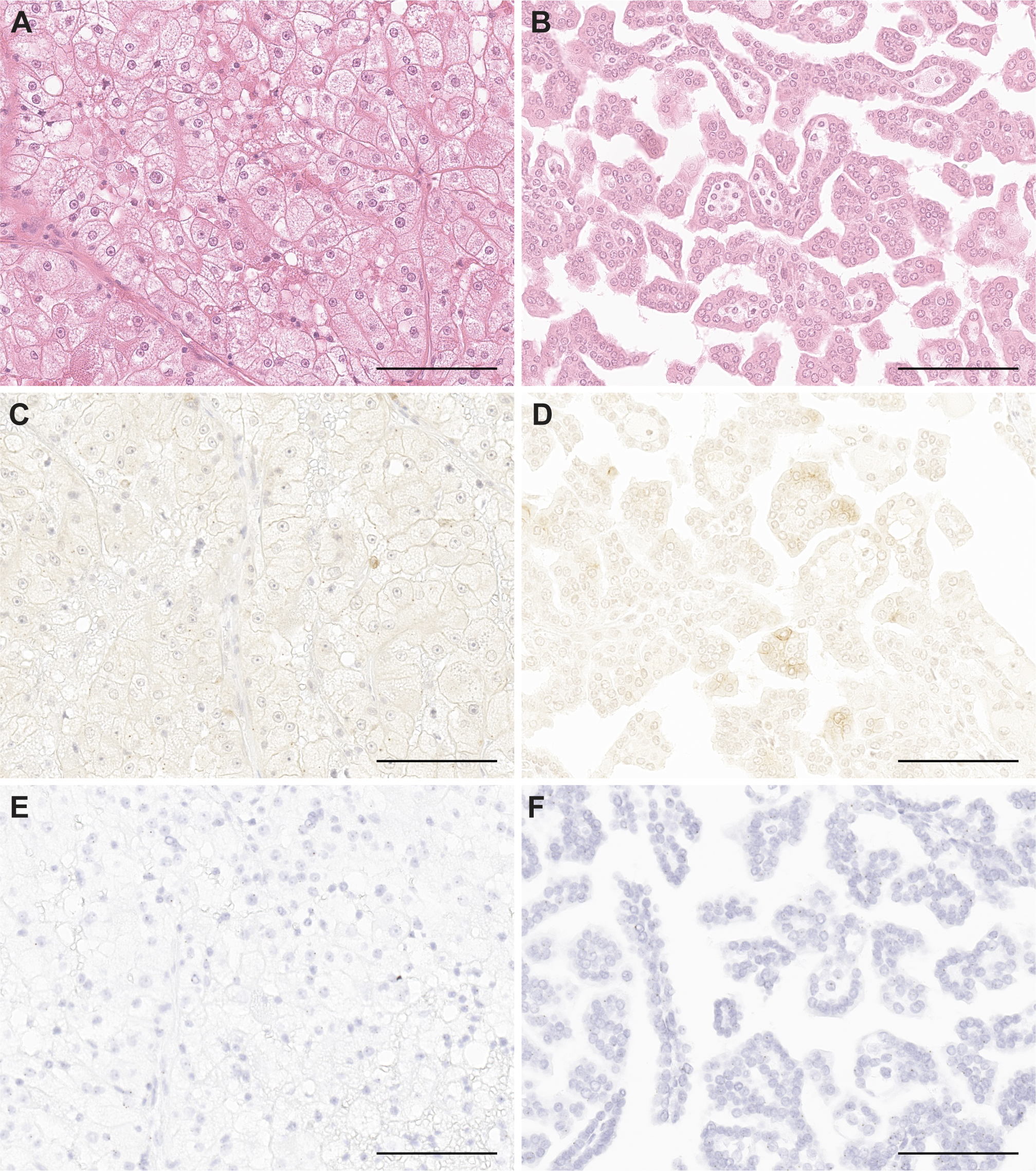

Supplement: Supplementary Figure 6 — L1CAM and LINC01187 expression in ccRCC and PRCC. ccRCC (A, H&E) demonstrated no expression of L1CAM (C) or LINC01187 (E). PRCC (B, H&E) showed no expression of L1CAM (D) in majority of evaluated cases and no expression of LINC01187 in all evaluated cases (F). Scale bars = 200μm. [file NIHMS1940187-supplement-Supplementary_Figure_6.tif]

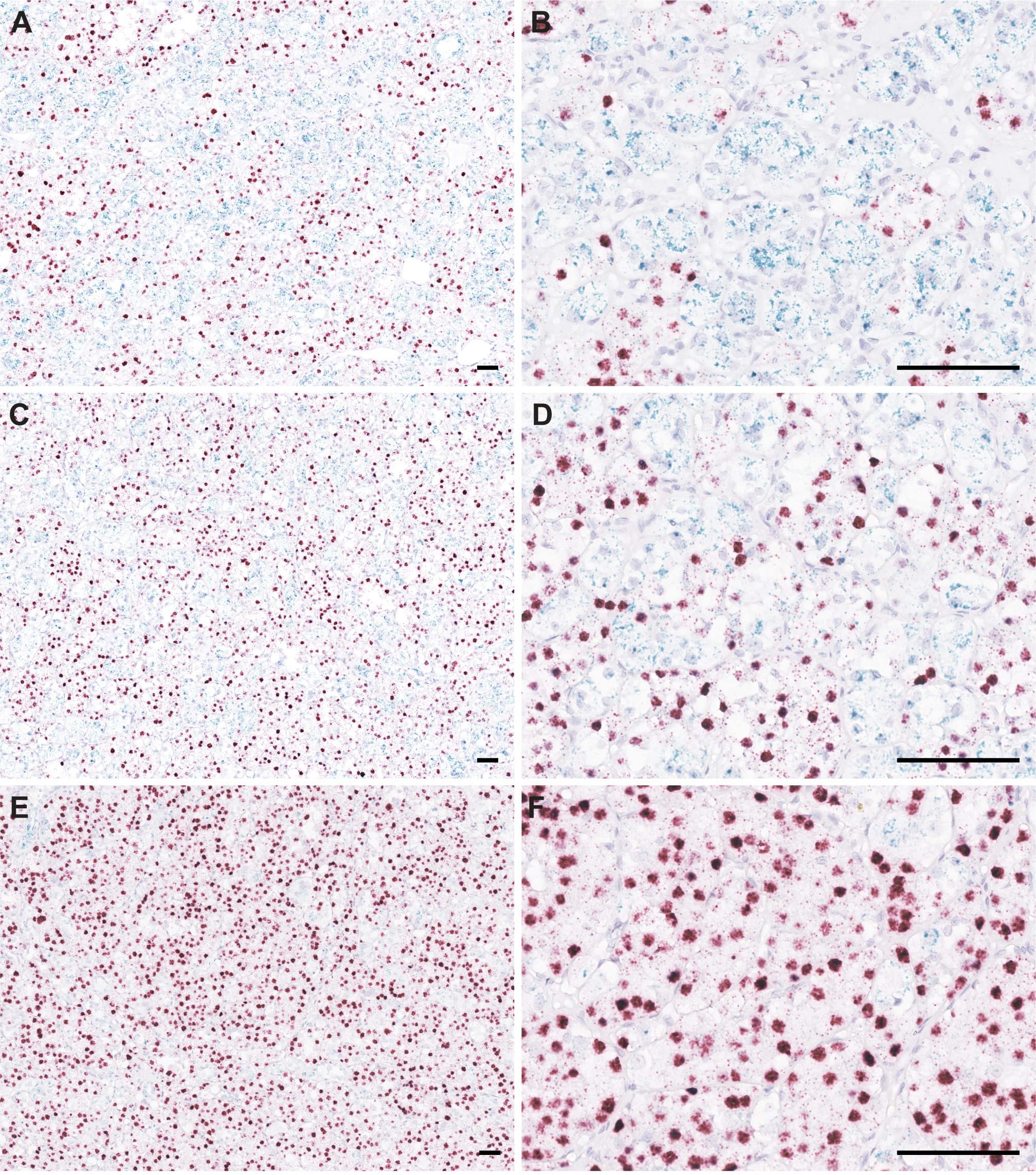

Supplement: Supplemental Data File (.doc, .tif, pdf, etc.)_2 — Supplementary Figure 7. Dual RNA-ISH for L1CAM (green signals) and LINC01187 (red signals) in a single tissue section from a HOT neoplasm demonstrated intra-tumor heterogeneity. Area 1 (A, B-zoom in), Area 2 (C, D-zoom in), and Area 3 (E, F-zoom in) showed variable proportions of neoplastic cells within HOT expressing L1CAM (green signals) or LINC01187 (red signals). Scale bars = 200μm. [file NIHMS1940187-supplement-Supplemental_Data_File___doc___tif__pdf__etc___2.tif]
